# Successful Treatment of a Left Main Thrombus by Intracoronary Eptifibatide Infusion in a 36-Year-Old Patient

**Published:** 2016-07-06

**Authors:** Saeed Alipour-Parsa, Elham Farahani

**Affiliations:** Cardiovascular Research Center, Shahid Beheshti University of Medical Sciences, Tehran, Iran.

**Keywords:** *Coronary vessels*, *Thrombosis*, *Eptifibatide*, *Therapeutics*

## Abstract

The acute coronary syndrome due to the left main coronary artery (LMCA) thrombosis is a clinically rare and catastrophic event. We describe a young man (smoker, alcoholic, and drug abuser) with a history of recent surgery and typical chest pain who had non-occlusive LMCA thrombosis in coronary angiography. The thrombosis was successfully treated with two 180 µ/kg intracoronary boluses of eptifibatide, which was continued through an intravenous infusion at 2 µ/kg/min for 48 hours postprocedurally. Control angiography, performed 3 days later, revealed that the LMCA was free of thrombosis. The patient had no complaints, including chest pain, and remained completely asymptomatic during the next 30 days' follow-up.

## Introduction

The acute coronary syndrome (ACS), including myocardial infarction (MI) (ST-segment elevation and depression, Q wave, and non-Q wave) and unstable angina, has evolved as a useful operational term to refer to any constellation of symptoms that are compatible with acute myocardial ischemia. The disruption of plaques is now considered the pathophysiological substrate of the ACS. When plaque disruption occurs, a sufficient quantity of thrombogenic substances (e.g. tissue factor) is secreted, and the coronary artery lumen may become obstructed by a combination of platelet aggregates, fibrin, and red blood cells.^1^

The ACS due to the left main coronary artery (LMCA) thrombosis is an unusual manifestation of coronary atheromatous disease.^2^ Since this artery supplies blood to the vast majority of the left ventricular myocardium, early recognition and emergent revascularization is vital for survival in this situation.

Coronary artery bypass grafting (CABG) and sometimes percutaneous coronary intervention (PCI) are the standard treatments for the unprotected LMCA disease. Several reports have recently opened a new therapeutic window for the use of a new promising class of antiplatelet medications as adjuncts to thrombolytic therapy in acute MI.^3^^, ^^4^

**Figure 1:**
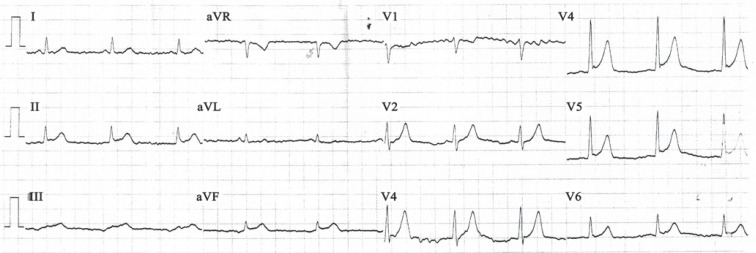
Patient’s ECG at presentation demonstrates diffuse hyper-acute T waves and ST changes in the precordial leads.

Eptifibatide, an anti-platelet agent with high affinity and specificity for the glycoprotein (GP) IIb/IIIa receptors, blocks platelet aggregation by inhibiting the binding of fibrinogen to activated platelet GP IIb/IIIa receptors, thereby inhibiting platelet–platelet interaction and thrombus formation.^5^

## Case Report

A 36-year-old man with a recent history of spinal surgery was referred to the Cardiac Emergency Department of Shahid Modarres Hospital in Tehran, Iran, with a typical chest pain of 3 hours' duration. The patient was a smoker, alcoholic, and substance abuser. He had used inhalational opium and crystal for the last 2 years. He had no known past medical history or familial history of coronary artery disease. His vital signs on admission were as follows: blood pressure of 150/100 mmHg; heart rate of 68 bpm; respiratory rate of 14/minute; oral temperature of 37.2 ^°^C; and oxygen saturation of 93% on room air. Physical examination was unremarkable except for a surgical wound at the lumbar region.

**Figure 2:**
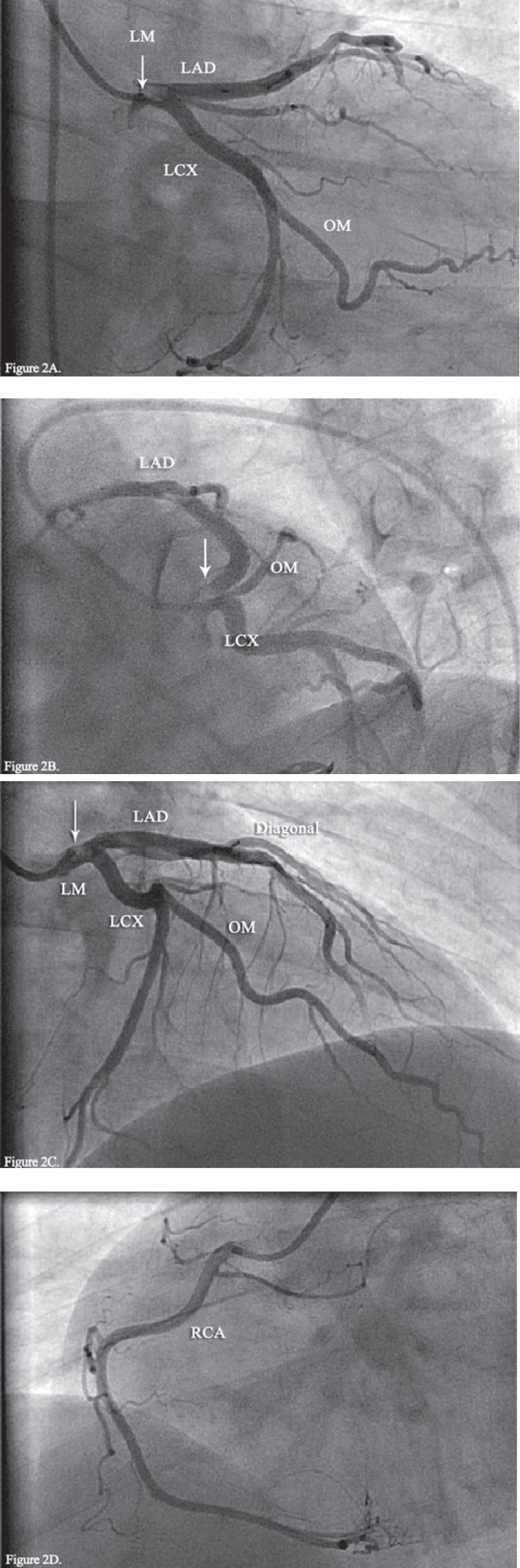
Coronary angiogram at presentation:

With the clinical impression of the ACS, a twelve-lead ECG was obtained, which revealed diffuse ST-segment elevations in V_2 _through V_6 _and inferior leads (Figure 1). Bed-side echocardiography revealed preserved left ventricular systolic function (ejection fraction = 50%), apical hypokinesia, and trivial mitral regurgitation with minimal pericardial effusion. Immediate coronary angiography showed normal right coronary artery, a thrombolysis in myocardial infarction II flow (TIMI II flow) in the distal part of the left anterior descending artery (LAD), and a large non-occlusive filling defect in the LMCA (Figure 2). The ejection fraction was about 45% on left ventricular angiography with apical hypokinesia.

(A) Anteroposterior (AP) caudal view shows a filling defect in the left main coronary artery (arrow).

(B) Left anterior oblique (LAO) caudal view shows partial obstruction of the left main trunk by a thrombus (arrow).

(C) Right anterior oblique (RAO) caudal view confirms the same thrombus with intact main branches (arrow).

(D) Right coronary artery (RCA) injection shows a normal right coronary artery.

LM, Left main coronary artery; LAD, Left anterior descending artery; LCX, Left circumflex coronary artery; OM, Obtuse marginal coronary artery; RCA, Right coronary artery

**Figure 3:**
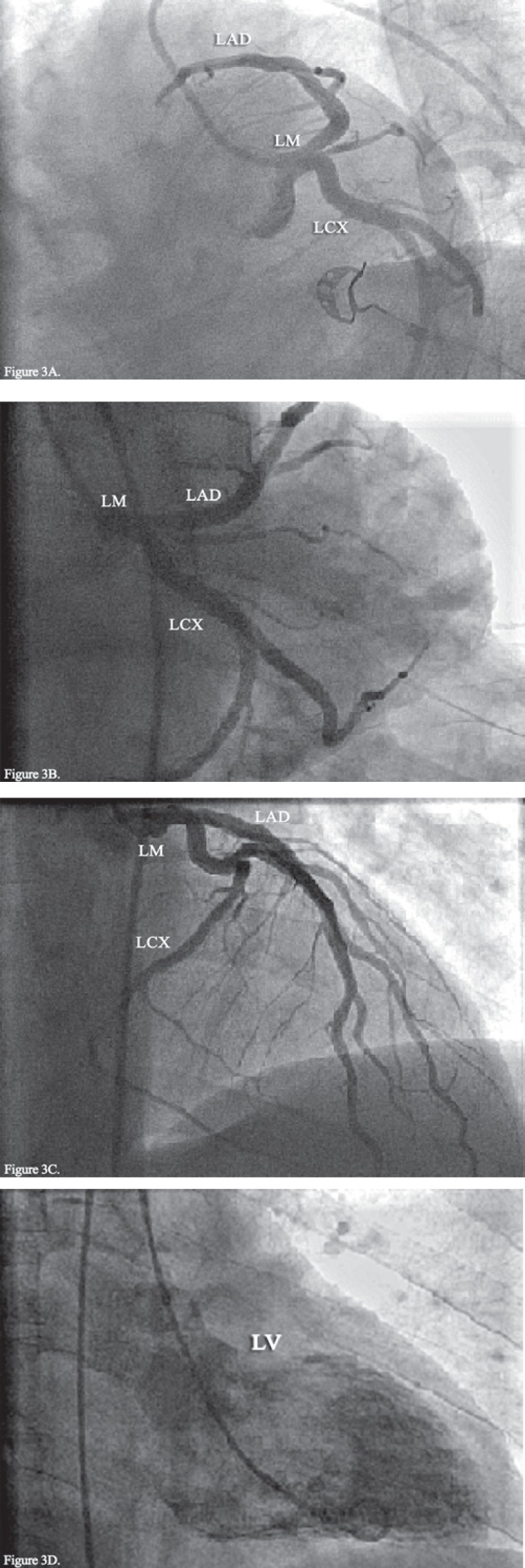
Control coronary angiography after 3 days of treatment with eptifibatide shows normal coronary arteries with the disappearance of the aforementioned filling defect in Figure 2 (A-C) and a normal left ventricular injection (D).

LM, Left main coronary artery; LAD, Left anterior descending artery; LCX, Left circumflex coronary artery; LV, Left ventricle

**Figure 4:**
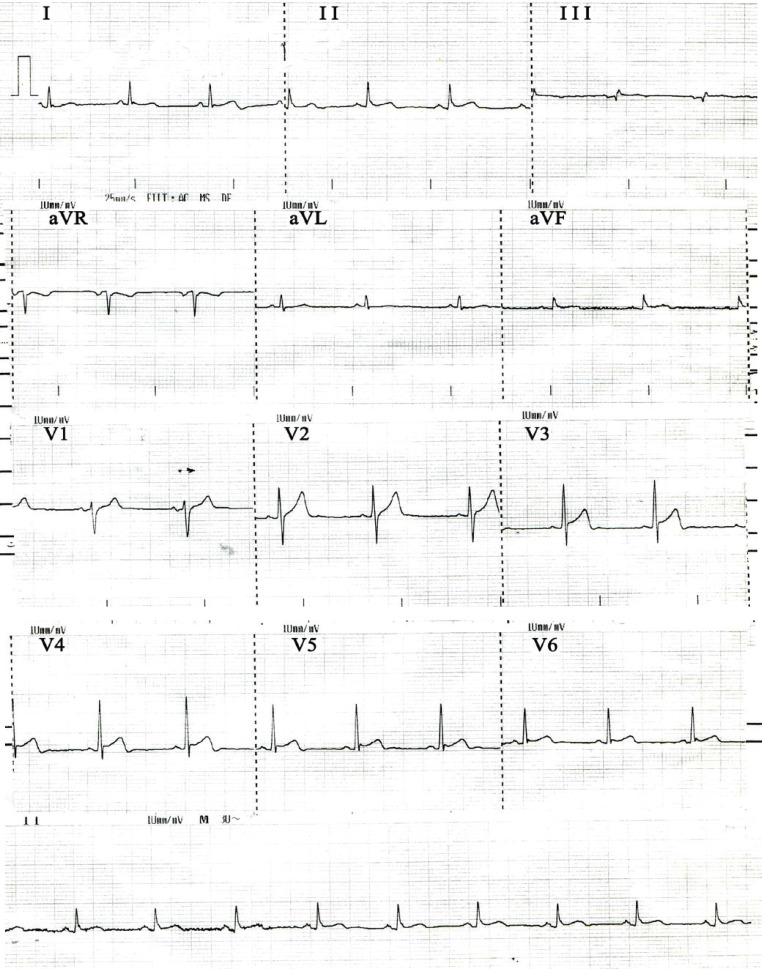
ECG after 3 days of treatment with eptifibatide shows the complete resolution of ST-T changes in the precordial leads.

Two 180 µ/kg intracoronary boluses of eptifibatide (Integrilin, COR Therapeutics, Inc., San Francisco, California) were injected, 10 minutes apart, through a 6-French extra back-up 3.5 (XB 3.5, Cordis Corporation) guiding catheter used for cannulating the left coronary ostium. The injections were continued through an intravenous infusion at 2 µ/kg/min for 48 hours postprocedurally. Additionally, 300 mg loading doses of Aspirin and Clopidogrel, 50 mg of Metoprolol, 12.5 mg of Captopril, and 80 mg of Atorvastatin were given and intravenous nitroglycerine 5 mic/min and Heparin infusion starting with 15 IU/kg/hr aiming at an activated clotting time of about 200-250 seconds were administered for the next 48 hours. Although routine cases of primary PCI are conducted in the setting of acute MI, we opted not to perform aspiration thrombectomy in our patient due to the ostial and floating nature of his thrombus and its unpredictable behavior, which could potentially lead to distal or even systemic embolization in this special situation.

Control angiography, performed 3 days later, revealed that the LMCA was free of thrombosis. In addition, the LAD had a TIMI III flow and there was no apical hypokinesia (Figure 3). At this point, the patient had no complaints, including chest pain. The ECG also showed normalization of the initial abnormalities (Figure 4). Nevertheless, laboratory tests demonstrated marked elevations in cardiac biomarkers: Creatinine kinase (CK)-MB was 132 and 165 mg/dl after 6 and 12 hours (normal range = up to 25 mg/dl) and troponin T was 1.81 and 3.39 ng/ml after 6 and 12 hours respectively (normal range < 0.06 ng/ml). Moreover, the patient had prothrombin time of 12 (normal range = 10–13 sec); partial thromboplastin time of 30 (normal range = up to 43 sec); white blood cell count of 11.9 × 10^3^/µL (normal range = 4–10×10^3^/µL); hemoglobin (Hb) of 12.7 g/dL (normal range = 11.8–17 g/dL); platelet count of 313×10^3^/µL (normal range = 150–450×10^3^/µL); creatinine of 1 mg/dL (normal range = 0.5–1.5 mg/dL); and negative hepatitis-B antigen, anti-hepatitis-C antibody, and anti-human immunodeficiency virus antibody.

Transesophageal echocardiography was performed and ruled out any patent foramen ovale as a possible source of thromboemboli. There was no minor or major bleeding at the site of surgery after treatment. The patient's 5 days' hospital course was favorable, and he was discharged with Aspirin, Clopidogrel, and statin. He was completely asymptomatic during the next 30 days' follow-up.

## Discussion

Acute LMCA thrombosis is a serious condition which can present as the ACS, ST-segment elevation myocardial infarction (STEMI), cardiogenic shock, and sometimes even sudden cardiac death.^6^ We present a rare case that manifested with the ACS due to the LMCA thrombosis.

The usual cause of the LMCA obstruction is atherosclerotic occlusion resulting from plaque rupture and subsequent thrombus formation. Other causes reported are embolism, aortic dissection, pulmonary artery compression, and vasospasm.^7^ Moreover, embolus to the LMCA can be seen in patients with prosthetic heart valves without evidence of a thrombus.^8^^-^^10^ Other etiologies of the LMCA occlusion include catheter-induced,^11^ cocaine-induced plaque rupture or spasm,^12^ mycotic aneurysms of the LMCA,^13^ extrinsic compression from the pulmonary artery,^14^ as a complication of radiofrequency ablation procedures involving the atrioventricular node,^15^ fibrous intimal proliferation after cardioplegia during bypass or valve replacement surgery,^16^ and blunt chest trauma.^17^

The goal of management in acute MI is restoration of the blood flow as quickly as possible, but there are no specific guidelines for managing the LMCA thrombosis.^18^ For many years, CABG and PCI have been regarded as the gold-standard treatment for the LMCA disease.^19^ By exploring the role of platelet activity in the development of the ACS,^20^^–^^23^ clinical trials have now opened a new therapeutic window for the use of platelet GP IIb/IIIa receptor blockers as an adjunct to thrombolytic therapy in acute MI.^3^^, ^^4^ Several trials in the field of non-ST-segment elevation ACS have demonstrated the benefits of GP IIb/IIIa receptor inhibitors used as an anticoagulant.^24^ Several large trials involving patients with unstable angina/ non-ST elevation myocardial infarction (NSTEMI) have shown that the GP IIb/IIIa inhibitors are of substantial benefit for patients at high risk, those undergoing PCI, or both.^25^^, ^^26^ Be that as it may, because of the different protocols and agents that were used and the relatively small number of the patients included in these studies, no definite conclusion can be drawn about the role of platelet GP IIb/IIIa receptor blockers in these circumstances. Nevertheless, one could also make a decision for using GP IIb/IIIa receptor blockers even as a monotherapy for acute MI in special situations, as in the aforementioned case.

Given that our patient had undergone a major surgical operation (spinal surgery) 5 days before he presented to us, there was relative contraindication for streptokinase infusion, which is the main thrombolytic agent in our region. Furthermore, there was no indication for PCI or CABG in our patient due to a lack of obvious stenosis and a TIMI II flow in the distal LAD in conjunction with a filling defect in the LMCA. Accordingly, after an injection of 5000 units of Heparin, we decided to give intracoronary and then a 48-hour intravenous infusion of eptifibatide in conjunction with Heparin infusion. The patient’s response was excellent and second angiography, performed 3 days subsequently, showed a TIMI III flow in the LAD without residual thrombosis in the LMCA.

## Conclusion

Alongside surgery and PCI, GP IIb/IIIa inhibitors might be used as a therapeutic option in clinical situations where thrombolytic agents are contraindicated and there is in situ thrombosis without stenotic lesions in the coronary arteries, especially the LMCA.

## Notes:

***This paper should be cited as:*** Alipour-Parsa S, Farahani E. Successful Treatment of a Left Main Thrombus by Intracoronary Eptifibatide Infusion in a 36-Year-Old Patient. J Teh Univ Heart Ctr 2016;11(3):143-148.
